# Rapid and simultaneous detection of common aneuploidies by quadruplex real-time polymerase chain reaction combined with melting curve analysis

**DOI:** 10.1371/journal.pone.0171886

**Published:** 2017-02-27

**Authors:** Jiwu Lou, Manna Sun, Ying Zhao, Zhisong Ji, Fenghua Liu, Dongzhi Li, Wanfang Xu, Yangyang Lin, Yanhui Liu

**Affiliations:** 1 Prenatal Diagnosis Center, Dongguan Maternal and Child Health Hospital, Dongguan, Guangdong, People's Republic of China; 2 Reproductive medicine Center, Guangdong Maternal and Child Health Hospital, Guangzhou, People's Republic of China; 3 Prenatal Diagnostic Center, Guangzhou Women & Children Medical Center affiliated to Guangzhou Medical University, Guangzhou, Guangdong, People’s Republic of China; Hospital Authority, CHINA

## Abstract

**Background:**

During the prenatal period, the number variation of chromosomes 13, 18, 21, X and Y accounts for more than 80% of the clinically significant chromosomal abnormalities diagnosed. Rapid tests for prenatal diagnosis of these abnormalities can improve pregnancy management and alleviate parental anxiety. Here, we present a molecular alternative method for detecting common aneuploidies.

**Methods:**

This method is based on co-amplification of segmental duplications located on two different chromosomes using a single pair of primers. Segmental duplications have a high degree of sequence identity, but have single-nucleotide differences in some regions. These sequence differences can be quantified using melting curve analysis of dual-labeled probes to estimate the relative dosages of different chromosomes. We designed two quadruplex real-time PCR assays to detect aneuploidies of chromosomes 13, 18, 21, X and Y.

**Results:**

We examined 75 aneuploid DNA samples and 56 unaffected DNA control samples using these two assays and correctly identified all samples. Four cases of unbalanced translocation were also accurately detected. The observed averaged ratio for each chromosomal disorder was similar to the theoretically expected value.

**Conclusions:**

Our real-time assay is a robust, rapid, and easy to conduct technique for prenatal diagnosis of common aneuploidies, representing a competitive alternative for use in diagnostic laboratories.

## Introduction

During the prenatal period, the number variation of chromosomes 13, 18, 21, X and Y account for more than 80% of the clinically significant chromosomal abnormalities diagnosed [1]. Karyotype analysis, a common diagnostic test for chromosomal abnormalities, has been regarded as the standard method for prenatal cytogenetic diagnosis since the early 1970s [2]. However, due to the long time required to obtain results (typically 2–4 weeks) and the risk of cell culture failure, the use of karyotype analysis in prenatal diagnosis is limited. With the widespread use of antenatal screening methods based on maternal serum analysis, fetal ultrasonography, and non-invasive prenatal testing (NIPT) for the more common aneuploidies of chromosomes 13, 18, 21, X and Y, a method for rapid, targeted detection of these aneuploidies may provide more benefit to pregnant women than conventional karyotype analysis [3–5].

In clinical practice, fluorescent in situ hybridization (FISH), quantitative fluorescence PCR (QF-PCR), and multiplex ligation-dependent probe amplification (MLPA) are the three most commonly used rapid, targeted aneuploidy detection methods [6]. All three methods avoid the generation of cultured cells and can rapidly detect (within 1 or 2 days) the most common aneuploidies (trisomy 13, 18, and 21) and aneuploidies of the sex chromosomes. However, these techniques have drawbacks. FISH is relatively labor intensive and requires technical expertise, which prevents the efficient processing of a large number of samples in a clinical diagnostic setting. Compared to FISH, QF-PCR and MLPA are more suited to a high throughput diagnostic service; they are PCR-based methods that involve the amplification of polymorphic microsatellite markers and ligated probes, respectively. Differences in the number of informative polymorphisms might limit the universality of QF-PCR. With MLPA, the potential problem of non-informativeness is eliminated, but an overnight step for hybridization lasting approximately 16 hours is required. Moreover, both QF-PCR and MLPA require a post-PCR step for quantitative analysis of the products using an expensive genetic analyzer.

Many real-time PCR-based methods without post-PCR steps have also been developed [7–13]. Among them, paralogous sequence quantification technique-based methods are most ideal because simultaneous amplification of paralogous genes or segmental duplication sequences using a single pair of primers can maintain the original ratio between the test chromosomes and reference chromosomes and avoid the dependence on short tandem repeat polymorphisms or SNPs. However, existing methods cannot simultaneously detect multiple targeted chromosomes in one tube, thus reducing sample throughput [11,12]. The purpose of this study was to develop a real-time PCR assay based on segmental duplication sequence amplification combined with melting curve analysis of 4-color, fluorescently-labeled probes. The assay was able to accurately and rapidly diagnose aneuploidy in chromosomes 13, 18, 21, X and Y in two PCR tubes, meeting the requirements for large-scale screening of chromosomal aneuploidies.

## Materials and methods

### Samples

Seventy-five DNA samples with a known aneuploid karyotype were used for this study. Of these samples, 28 were obtained from individuals with trisomy 21 (27 uncultured amniotic fluid samples and 1 CVS sample); 9 were obtained from individuals with trisomy 18 (8 uncultured amniotic fluid samples and 1 CVS sample); 7 were obtained from individuals with trisomy 13 (6 CVS samples from aborted fetuses and 1 uncultured amniotic fluid sample); 15 were obtained from 45,X individuals (14 CVS samples from aborted fetuses and 1 uncultured amniotic fluid sample); 8 were obtained from 47,XXX individuals (8 uncultured amniotic fluid samples); 4 were obtained from 47,XXY individuals (4 uncultured amniotic fluid samples); and 4 were obtained from 47,XYY individuals (4 uncultured amniotic fluid samples). The samples obtained from 56 unaffected individuals consisting of 32 females and 24 males (54 uncultured amniotic fluid samples and 2 CVS samples) were used as unaffected controls. Four additional samples with unbalanced translocations (46,XX,del(18)(p10), 46,XX,del(18)(q10), 46,X,i(X)(q10) and 45,X [12]/45,X,+mar[12]) were also included. These samples were collected from pregnant women referred for fetal genetic analysis at Dongguan Maternal and Children's Hospital between January 2014 and February 2016. Written informed consent was obtained from the (pregnant) mother of each subject. Samples were archived in our laboratory, and researchers had no access to information that could identify individual subjects during the study from March 2015 to Sep 2016. The Research Ethics Committee of Dongguan Maternal and Children's Hospital approved this study protocol. The concentration of extracted genomic DNA was determined using a NanoDrop 2000 spectrophotometer (Thermo Scientific, Boston, USA).

### Segmental duplications and primers/probes

The segmental duplication sequences were obtained from two public databases: the Segmental Duplication Database (http://humanparalogy.gs.washington.edu/) and the NCBI (http://www.ncbi.org/). We selected eight segmental duplications as molecular markers for the qPCR assay that met the following criteria: (1) only two copies on the human genome (one on a test chromosome and one on another reference chromosome) and (2) high sequence identity, but with sequence differences in one nucleotide observed in some regions. Eight PCR primer pairs selected from regions with identical sequences were used to amplify the eight segmental duplication sequences. Eight dual-labeled probes were designed to detect the nucleotide differences. The sequences of primers and probes are listed in Table 1. The Tm for probe hybridization with and without mismatches was predicted using Oligo Analyser (http://www.idtdna.com/calc/analyzer).

**Table 1:** Information for primers and probes.

Primer pair and probe	Chromosome location	F primer	R primer	Probe
Autosome				
12/21	12p13.11/21q21.22	AGTGCAAGTTCTAGCTCTGTTGA	TTCATTGGTTAGCAGCTGGTTC	FAM-CGAGAGATGCTCACCAA***A***CTCCCA-BHQ1
13/18	13q21.33/18p11.22	ATCCAGACTGTTCAGACCATCA	GTTCAAACTTGGCCTGCACCT	HEX-CAAGTGTCTCAC***G***CCAAAGGCT-BHQ2
13/21	13q31.3/21q21.22	TGGGACATGCTTCTGGTTAGTG	ACATGGTTGAGGCTGAGCAGTGAG	ROX-CTTACATCAAA***G***GCAGCTCTTCT-BHQ1
9/18	9p24.1/18q21.33	GAGCTGCGACACGGAGAAG	CAACCACACCTGCTGTTCA	CY5-CTGGCTGGTCCTC***G***TCTGCT-BHQ2
Sex chromosome				
16/X	16q12.1/Xp11.23	ACAGAGCCTGCATGGAAGA	AGCCTGAAGTCAGTGGAGA	FAM-TGAATCATTG***G***TCAGGACATC-BHQ1
X/Y	Xp22.33/Yq11.21	CTAAAGGAAGGCAGTGTTTGTTA	GACCTATCAGTCTTCAGCTTGTC	HEX-CATGTAGAGAA***G***GATAATGTCTG-BHQ2
3/X	3p24.2/Xq21.3	TAACTGACCCAAAACTACCTGTC	TGCCTAATGTTTTGTGATTCACT	ROX-ATGTCAGA***G***CAGTTGGGT-BHQ1
X/Y	Xq21.3/Yp11.2	AAGACAGCCCGGCGAAGA	ATTCCGGGAGAATGCGTCTG	CY5-TCTTTGTTGC***A***GATTTCTAACTGGT-BHQ2

As described in detail previously [14], for the detection of sex chromosome abnormalities, the copy number status of X was first obtained using X/autosome primer pairs and probes, and the copy number status of Y was then quantified using X/Y primer pairs and probes.

### Real-time PCR

Two quadruplex reactions were designed to evaluate the method. One “one-tube test” was used for chromosomes 13, 18 and 21, and another “one-tube test” was used to screen for sex chromosome aneuploidies. Asymmetric PCR was performed in a final volume of 20 μL containing 50–100 ng of genomic DNA, 100 mM of each dNTP, 0.4 μl of Phire Hot Star II DNA Polymerase (Thermo Scientific, USA), 4 μL of 5×PCR buffer, 0.25 mM of each dual-labeled probe, 0.1 mM of each forward primer, and 1 mM of each reverse primer. PCR was performed using a SLAN-96S Real-time PCR system (Hongshi Medical Technology Co., Ltd, Shanghai, China) with an initial denaturation step at 95°C for 5 min, followed by 50 cycles of denaturation at 95°C for 20 s, annealing at 60°C for 20 s, and elongation at 72°C for 20 s. The melting program consisted of 95°C for 1 min, 35°C for 5 min, and 80°C for 30 s. The rate of temperature increase was 0.5°C/step with a 20 s pause between each step.

### Data analysis

**Fig 1:**
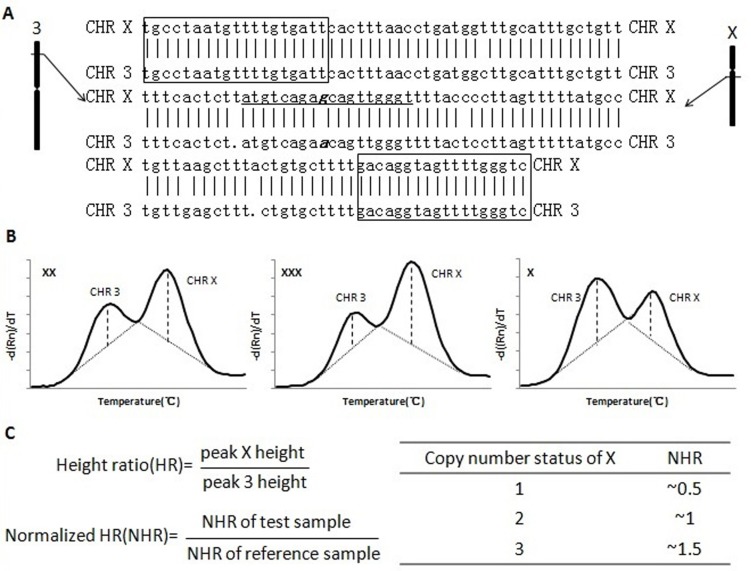
Principles of melting analysis for aneuploidy (example presented for the X chromosome). (A) Typical alignment between segmental duplication sequences used to design primers and probes. Rectangular boxes indicate the positions of primers; the underlined letters indicate the probe sequences, and the bold and underlined letters indicate sequence mismatches used for quantification of melting analysis. (B) Melting curve patterns for various copy numbers of X. Dotted lines indicate the peak baseline, and vertical dashed lines indicate the peak height. (C) Principles of data analysis.

After amplification and melting curve analysis, the peak heights under the melting curves from the test and reference chromosomes were determined by the SLAN 8.2 software and exported to an Excel spreadsheet. The peak height ratio (HR) was calculated by comparing the test chromosome peak height to the reference chromosome peak height. The peak HR of each experimental sample was normalized to that of the control samples. In theory, the normalized peak height ratio (NHR) should be ~1.0 for normal disomy and~1.5 and ~0.5 for trisomy and monosomy, respectively (Fig 1, Tables 2 and 3 and S1 Table). In addition, under normal conditions, the melting curve pattern of a sample without genomic abnormalities, in each channel, will be identical to that of reference samples. If the conclusions from NHR and melting curve pattern are obviously inconsistent, a custom Excel macro is used to recalculate the peak heights and NHR (S1 Fig and S1 Text).

**Table 2:** Observed NHRs for chromosomes 21/18/13 in the four channels.

Type of aneuploidy	Case number	FAM(12/21)	HEX(18/13)		ROX(21/13)		CY5(9/18)
		21	18	13	21	13	18
control	56	1.00±0.04*	1.00±0.05	1.00±0.05	1.00±0.04	1.00±0.04	1.01±0.07
		(0.91–1.12)*	(0.84–1.11)	(0.90–1.19)	(0.90–1.09)	(0.91–1.11)	(0.88–1.19)
Trisomy 21	28	1.51±0.08	1.02±0.04	0.98±0.04	1.49±0.11	0.67±0.04	1.01±0.07
		(1.37–1.76)	(0.91–1.11)	(0.90–1.10)	(1.37–1.85)	(0.54–0.73)	(0.83–1.13)
Trisomy 18	9	1.09±0.03	1.34±0.06	0.75±0.03	1.01±0.07	0.99±0.08	1.53±0.12
		(1.03–1.15)	(1.26–1.46)	(0.68–0.80)	(0.86–1.13)	(0.88–1.16)	(1.35–1.68)
Trisomy 13	7	1.01±0.04	0.73±0.02	1.38±0.04	0.71±0.03	1.43±0.05	0.91±0.11
		(0.93–1.06)	(0.70–0.76)	(1.31–1.43)	(0.66–0.75)	(1.36–1.50)	(0.73–1.06)

*The NHRs of chromosome 21 in the FAM channel in four normal cases were incompatible with melting curve patterns. The peak heights were recalculated using the macro, and the new NHRs were consistent with the melting curve patterns and were used for statistical analysis (see S1 Table).

**Table 3:** Observed NHRs for sex chromosomes in the four channels.

Type of sex chromosome	Number	FAM(16/X)	HEX(3/X)	ROX(X/Y)	CY5(X/Y)
		X	X	Y	Y
XY	24	0.51±0.05 (0.45–0.65)	0.46±0.05 (0.39–0.62)	1.01±0.06 (0.85–1.09)	0.96±0.03 0.91–1.03)
XXY	4	0.95±0.18 (0.75–1.19)	1.08±0.03 (1.05–1.12)	0.59±0.04 (0.55–0.63)	0.51±0.02 (0.49–0.53)
XYY	4	0.48±0.03 (0.46–0.52)	0.48±0.03 (0.44–0.51)	1.73±0.14 (1.57–1.83)	1.92±0.24 (1.63–2.08)
XX	32	0.97±0.06 (0.84–1.13)	0.99±0.09 (0.88–1.19)	--	--
X0	15	0.52±0.09 (0.43–0.71)	0.50±0.06 (0.41–0.65)	--	--
XXX	8	1.43±0.12 (1.30–1.63)	1.41±0.11 (1.32–1.63)	--	--

The peak height ratio of X was normalized with that of normal female samples. The peak height ratio of Y was normalized with that of normal male samples.

### Classification criteria

We originally pre-screened a panel of 56 control samples using an autosomal assay. As observed in the MLPA assay, the normalized peak height ratio (NHR) in each channel was similar to the theoretically expected value. Thus, in the rest of the blind experiments, we distinguished various types of karyotypes according to classification criteria modified from those of the MLPA assay, as follows: (1) Monosomy: the mean of NHRs from two primer pairs and probes is <0.7, with both NHRs <0.8; (2) Normal results (identical to the reference result): the mean of NHRs is 0.8~1.2, with one 0.7~1.3 and another 0.8~1.2. For the 18/13 and 13/21 primer pairs and probes, the NHR for a normal result is 0.62~0.8 (0.8/1.3~1.2/1.3) if the copy number of the reference chromosome is 3 and the mean NHR is 0.71~1.1; (3) Trisomy: the mean of NHRs is >1.3, with one >1.3 and another>1.2; (4) XYY: the mean of NHRs for Y is >1.7, with both NHRs >1.5. (5) All other values were identified as uncertain result.

### Sensitivity analysis

The duplex reaction was performed using 10-fold serially diluted genomic DNA (between5 pg and 500 ng per reaction mixture) extracted from trisomy 13, trisomy 18, trisomy 21, 45,X and normal female samples. Water was used as the no template control.

## Results

Using melting curve analysis, the segmental duplication sequences located on both the test and reference chromosomes were amplified, and then the PCR products were subjected to melting curve analysis. Two distinct melting curve peaks were generated from the single nucleotide difference in the two sequences. Because the segmental duplication sequences were amplified by a single pair of primers, in theory, the original ratio of the two sequences should be preserved by the PCR products and would be further reflected in the ratio of the two melting curve peak heights (Fig 1) [11,14,15]. Probes could be labeled with different fluorescent dyes; therefore, different chromosomes could be simultaneously detected in a “one-tube test”. We designed two quadruplex PCR assays to detect trisomies of chromosomes 13, 18 and 21 and sex chromosome aneuploidies. Each assay contained four primer sets and four corresponding probes to ensure that each test chromosome could be simultaneously detected by two primer pairs and probes.

**Fig 2:**
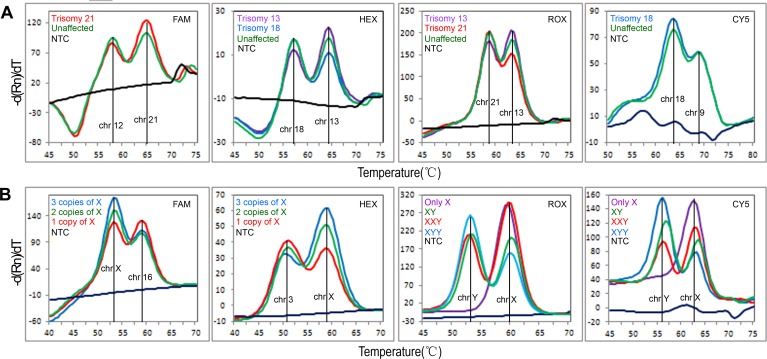
Melting curve patterns for trisomy of chromosomes 13, 18 and 21 (A) and sex chromosome aneuploidies in the four channels (B). NTC indicates a no-template control. The melting curve pattern and corresponding legend are labeled with the same color.

Using these two assays, we examined 75 aneuploid DNA samples and 56 unaffected DNA controls (from 24 males and 32 females). The observed mean NHR for each chromosomal disorder in each channel was similar to the theoretically expected value, with no region of overlap in mean±2S. We correctly identified all the samples according to the classification criteria. The typical melting curve patterns and NHR means are shown in Fig 2 and Tables 2 and 3, respectively.

Four cases of unbalanced translocation (46,XX,del(18)(p10), 46,XX,del(18)(q10), 46,X,i(X)(q10) and 45,X [12]/45,X,+mar[12]) were also accurately detected with these two assays. Except for chromosomes 13 and 21, every chromosome was tested by two primer pairs and corresponding probes for the short and long arms, respectively. In the case of the 18p deletion, the NHRs of 18p and 18q were 0.45 and 0.98, respectively. In contrast, the 18q deletion showed NHRs of 18p and 18q of 1.02 and 0.45, respectively. For 46, X, i(X)(q10), the NHRs of Xp and Xq were 0.45 and 1.55, respectively. The results of our assays of these three cases were consistent with the karyotypes. In the fourth case, the NPR of X indicated a single copy of X. However, the NHR of Yq was 0.58, indicating that the marker chromosome originated from the Y chromosome. We confirmed this result using an MLPA P095 aneuploidy kit.

The sensitivity test results demonstrated that the trisomy 13, trisomy 18, trisomy 21, 45,X and normal female samples could be correctly distinguished using samples ranging from 5 to 500 ng. The NHR was difficult to interpret or the melting peaks were not evident in a range of 0.05 to 0.5 ng per reaction mixture, indicating that the limit of detection was at least 5 ng of gDNA per reaction.

## Discussion

In this study, we present the melting curve analysis method as an alternative approach for the rapid and efficient detection of targeted aneuploidies. Compared to commonly used methods, including FISH, QF-PCR and MLPA, our assay was simple and fast; the experiment did not require post-PCR steps and could be performed automatically using a real-time PCR machine, and the diagnosis results can be available within 4 hours. A retrospective study of 75 positive samples and 56 normal samples showed that our assay could identify autosomal trisomies of chromosomes 13, 18, and 21 and sex chromosome number abnormalities with 100% accuracy. The observed average NHR in each channel for each chromosomal disorder was very similar to the theoretically expected value. This study showed that co-amplification of segmental duplication sequences with a single pair of primers that match perfectly at both loci resulted in almost identical amplification efficiencies and end point measurements using the melting curve analysis method. Therefore, this technique is quantitative, reliable, and consistent with previously published results (11, 14, 15).

Four samples with unbalanced translocations were also examined. Changes in the NHRs of primer pairs and probes located in the positions of unbalanced fragments were observed. This suggests that when a test sample produces inconsistent results in the two primer pairs and probes tested, structural abnormalities should be considered, and full karyotyping, FISH, or array comparative genomic hybridization analysis should be conducted. However, although not seen in our study, SNPs or CNVs within the targeted regions of the designed primers and probes may produce ambiguous or even false negative/positive results, which has been previously reported [11]. To resolve this problem, we analyzed two segmental duplications (sufficiently distant from each other) per chromosome using two independent primer sets and probes. This measure can reduce the probability of encountering SNPs or CNVs in the two segmentally duplicated regions at the same time, thus producing false negative or false positive results.

Aneuploidy identification using melting curve analysis has been previously reported [9,11,13,16,17]. The earliest methods were based on allele quantification combined with melting curve analysis of SNP loci. Similar to QF-PCR, this approach requires the selection of multiple SNP markers to produce an informative result for a chromosome. Thus, it is impossible to simultaneously detect multiple targeted chromosomes in one tube. Two other methods are based on HRM analysis of segmental duplications (SD-HRM) and competitive PCR with limiting dNTPs and high-resolution melting. In the SD-HRM approach, segmental duplications are also used, but HRM is applied to analyze the PCR products. To identify the aneuploidies of chromosomes 21, 18, 13, X and Y in a sample, 8–10 separate PCR reactions for each sample are required. Furthermore, this assay indicates only the relative signal difference rather than the relative copy number. Competitive PCR with limiting dNTPs and high-resolution melting does not require the fortuitous presence of homologous sequences to identify a common primer pair. This method is useful for targeted evaluation of relative copy numbers, including the confirmation of copy number changes that are revealed by microarrays or massively parallel sequencing. However, for HRM, distinguishing more than 4 different sequences with well-separated melting temperatures in one tube is difficult. With our assay, 8 different sequences could be distinguished in one tube, ensuring that each test chromosome could be simultaneously detected twice.

In conclusion, this method can automatically process large numbers of samples in a real-time PCR machine. It has also proven to be a simple and rapid alternative approach for simultaneous detection of chromosome 21, 18, 13, X and Y aneuploidies in a single reaction.

## Supporting information

S1 Fig Simple principle of determining the peak height using the custom macro. The key is to determine the co-ordinates of seven points (A, B, C, D, E, F and G) in the curve. A and G are the starting point and ending point of the curve, respectively; C and E are peak points; D is the lowest point between C and E; B is located between A and C, with the longest distance to line AC; F is located between E and G, with the longest distance to line EG. Lines BD and DF are the baselines of the two peaks, respectively. Peak height is the distance from the peak point to the intersection between the baseline and vertical line passing through the peak point.
(TIF)

S1 Table Data tables of all 75 aneuploid and 56 unaffected samples, including two types of tables (1). The NHR data table contains the Tm and peak height values determined using SLAN software and the Excel macro, respectively. NHRs are also listed in these tables. These files were named using “height” (2). Melting curve data tables. These tables contain the primary data for melting curves and were named using “chr”. In these tables, the first column indicates the plate wells consistent with the information in the NHR data table; the first row indicates the temperature.
(RAR)

S1 Text Code files of the custom macros used for autosomes and sex chromosomes, respectively. These code files can be executed in the melting curve data table to determine peak heights.
(RAR)
