# Supplementary figures and images for: Rapid and simultaneous detection of common aneuploidies by quadruplex real-time polymerase chain reaction combined with melting curve analysis
Source: PLoS One. 2017 Feb 27;12(2):e0171886. doi: 10.1371/journal.pone.0171886 (PMC5328249; doi:10.1371/journal.pone.0171886)

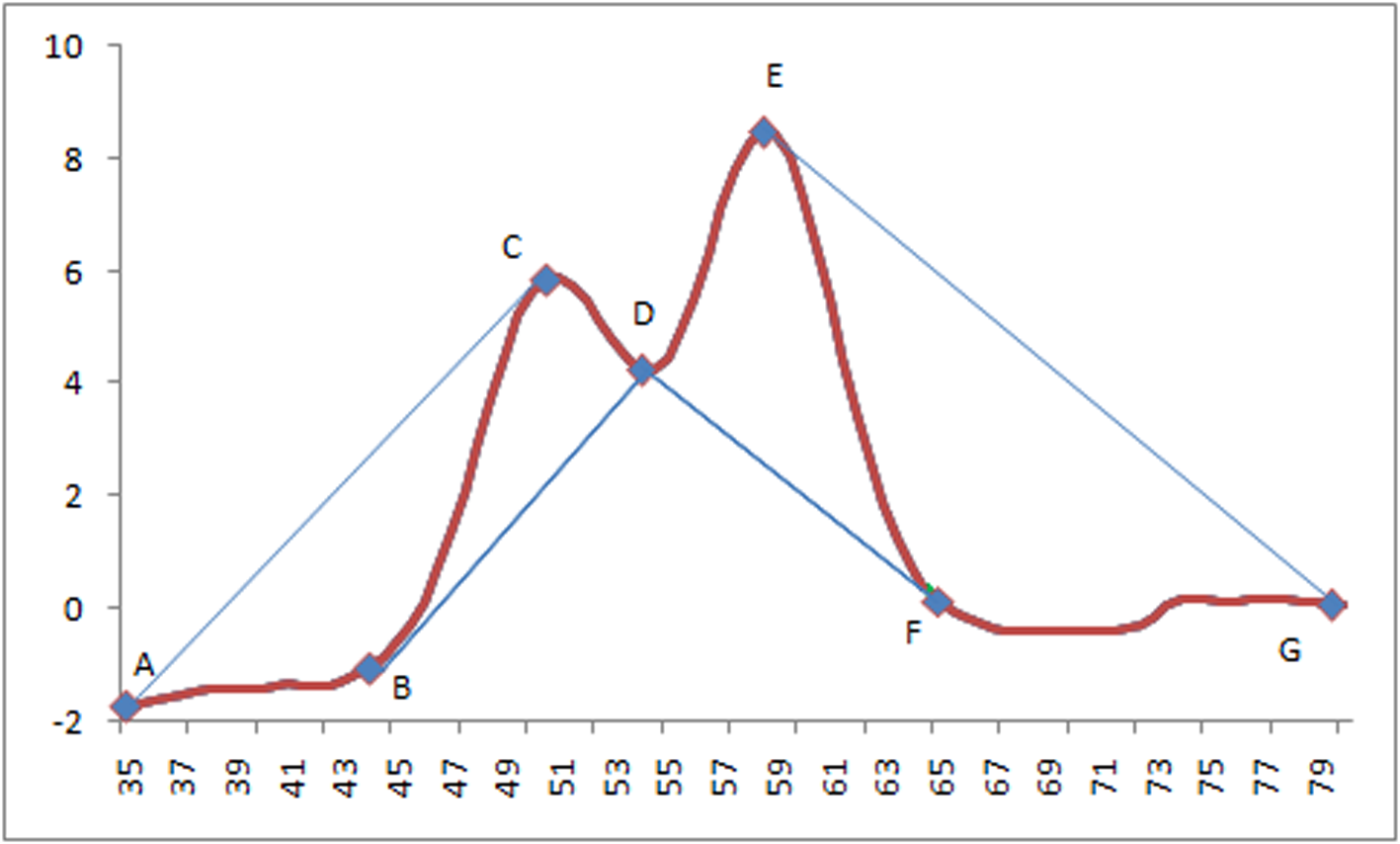

Supplement: S1 Fig — The key is to determine the co-ordinates of seven points (A, B, C, D, E, F and G) in the curve. A and G are the starting point and ending point of the curve, respectively; C and E are peak points; D is the lowest point between C and E; B is located between A and C, with the longest distance to line AC; F is located between E and G, with the longest distance to line EG. Lines BD and DF are the baselines of the two peaks, respectively. Peak height is the distance from the peak point to the intersection between the baseline and vertical line passing through the peak point. (TIF) [file pone.0171886.s001.tif]
